# Injectable, Pore‐Forming, Perfusable Double‐Network Hydrogels Resilient to Extreme Biomechanical Stimulations

**DOI:** 10.1002/advs.202102627

**Published:** 2021-11-22

**Authors:** Sareh Taheri, Guangyu Bao, Zixin He, Sepideh Mohammadi, Hossein Ravanbakhsh, Larry Lessard, Jianyu Li, Luc Mongeau

**Affiliations:** ^1^ Department of Mechanical Engineering McGill University Montreal QC H3A 0C3 Canada; ^2^ Department of Biomedical Engineering McGill University Montreal QC H3A 2B4 Canada

**Keywords:** cytocompatible, injectable, microfluidics, perfusable, porous structures, tissue engineering, tough hydrogels

## Abstract

Biological tissues hinge on blood perfusion and mechanical toughness to function. Injectable hydrogels that possess both high permeability and toughness have profound impacts on regenerative medicine but remain a long‐standing challenge. To address this issue, injectable, pore‐forming double‐network hydrogels are fabricated by orchestrating stepwise gelation and phase separation processes. The interconnected pores of the resulting hydrogels enable direct medium perfusion through organ‐sized matrices. The hydrogels are amenable to cell encapsulation and delivery while promoting cell proliferation and spreading. They are also pore insensitive, tough, and fatigue resistant. When tested in biomimetic perfusion bioreactors, the hydrogels maintain physical integrity under prolonged, high‐frequency biomechanical stimulations (>6000 000 cycles at 120 Hz). The excellent biomechanical performance suggests the great potential of the new injectable hydrogel technology for repairing mechanically dynamic tissues, such as vocal folds, and other applications, such as tissue engineering, biofabrication, organs‐on‐chips, drug delivery, and disease modeling.

## Graphical abstract

A design and methodology to form injectable, in situ pore‐forming, tough, and cytocompatible hydrogels are reported. Such hydrogels combine superior permeability and toughness, enabling direct medium perfusion through organ‐sized matrices. They are immune against extreme biomechanical loads and auspicious to cells. This work advances the injectable hydrogel technology and opens new opportunities in tissue engineering, drug/cell delivery, biofabrication, microfluidics, and organs‐on‐chips.

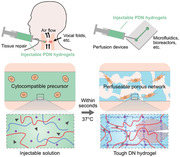

## 1 Introduction

Injectable hydrogels can be delivered via needle–syringe injection into the human body with low invasiveness.^[^ ^1^, ^2^ ^]^ They have found significant use in many branches of medicine, including drug/cell delivery, tissue engineering, biofabrication, organs on chips, and disease modeling. Despite extensive efforts in the field, there still remain challenges concerning the mass transport and mechanical properties of injectable hydrogels. On the one hand, most injectable hydrogels in use or under development are not perfusable due to their nanoporous structures.^[^ ^3^ ^]^ This issue limits the rapid transport of oxygen and nutrients (diffusion depth ≈ 600 µm), as well as the trafficking of native or transplanted cells.^[^ ^4^ ^]^ The adverse effects can also be found in microfluidics, such as organs‐on‐chips.^[^ ^5^ ^]^ Direct injection of hydrogels into microchannels tends to block the perfusion channel and thereby disable the devices. Immediate vascularization is therefore imperative but difficult to realize through injection. On the other hand, many injectable hydrogels cannot sustain large deformations and are susceptible to fracture in mechanically dynamic tissue environments.^[^ ^6^ ^]^ An extreme case is the vocal folds, arguably the most mechanically dynamic organ in the human body. Implants in the vocal fold lamina propria are exposed to up to 50% strains at a fundamental frequency that is on the order of 10^2^ Hz.^[^ ^7^ ^]^ Currently, patients with vocal fold injuries suffer from repeated hydrogel injections, due in part to the fracture‐induced short lifetime of the hydrogel implants under the dynamic loadings.^[^ ^8^ ^]^ By contrast, many biological tissues are perfusable and yet tough to tolerate extreme biomechanical stimulations as part of their normal functions, as exemplified by the vocal fold and heart.^[^ ^9^, ^10^ ^]^ To close the gap between injectable hydrogels and biological tissues, strategies to achieve a combination of high permeability and mechanical toughness are highly desired.

In past works, the incorporation of porous structures with injectable hydrogels has been realized to enable medium perfusion in lieu of vascularization. Those porous structures can be preformed before injection or formed in situ after placement in the human body. Preformed porous hydrogels can collapse to pass through a needle, and then regain their shape post‐injection.^[^ ^11^, ^12^, ^13^ ^]^ To prevent damage to such hydrogels during injection, the use of oversized needles is necessary but it increases the invasiveness of the procedure. In addition, the preformed method usually demands lengthy fabrication processes, such as cryogelation, lyophilization, or 3D printing, as well as prior knowledge about the shape and volume of the injection site to conform to the often irregular wound. Alternatively, in situ pore‐forming hydrogels are preferable as they can be delivered in liquid form through small‐sized needles and undergo a sol–gel transition into porous scaffolds in the human body. Such hydrogels have been developed in previous studies using either porogen,^[^ ^14^, ^15^ ^]^ leachable particles,^[^ ^16^ ^]^ nanoclay,^[^ ^17^ ^]^ granular particles,^[^ ^18^ ^]^ self‐assembly,^[^ ^19^, ^20^ ^]^ or polymer degradation.^[^ ^21^ ^]^ Bioprintable pore‐forming formulas, such as aqueous two‐phase emulsion system^[^ ^12^, ^22^, ^23^ ^]^ and hydrogel microstrands,^[^ ^24^ ^]^ could be used for injection as well. These hydrogels, however, suffer from limited pore size and low interconnectivity, which impair permeability and performance. Attempts to promote perfusion by enlarging the pores deteriorate their mechanical strength because pores essentially act as defects or cracks. This issue is particularly critical when the hydrogels are subjected to the biomechanical stimulations present in mechanically dynamic organs or tissues.

Circumventing the inverse correlation between porosity and toughness while ensuring injectability and cytocompatibility proves to be a challenge. Recently, a variety of double‐network (DN) hydrogels have been reported to tolerate large defects and pores thanks to their high fracture toughness.^[^ ^25^ ^]^ The pore insensitivity is attributed to the synergy of dissipative and stretchy networks, which are often denoted as “primary” and “secondary” networks, respectively. These strategies, however, cannot meet other above‐mentioned requirements for injectable perfusable hydrogels. While the dissipative primary network can be realized with biopolymers at mild conditions, there are concerns about the employment of the stretchy secondary network, which requires toxic precursors and/or harsh reaction conditions. To render the hydrogel precursor cytocompatible, synthetic and naturally derived polymers have been used as the secondary network components, including clickable polyethylene glycol, methacrylated hyaluronic acid, 4‐carboxyphenylboronic acid‐grafted poly(vinyl alcohol), and polyacrylamide‐*co*‐diacetone acrylamide.^[^ ^26^, ^27^, ^28^, ^29^, ^30^ ^]^ The resulting tough hydrogels are nonetheless nanoporous. Naturally derived fibrous networks, such as platelet‐rich fibrin, have also been explored to form the secondary network but at the expense of significantly reduced stretchability.^[^ ^31^ ^]^ It has also been shown that a high toughness often restricts activities for the cells that reside within and hinders their cellular functions.^[^ ^32^ ^]^

**Figure 1:**
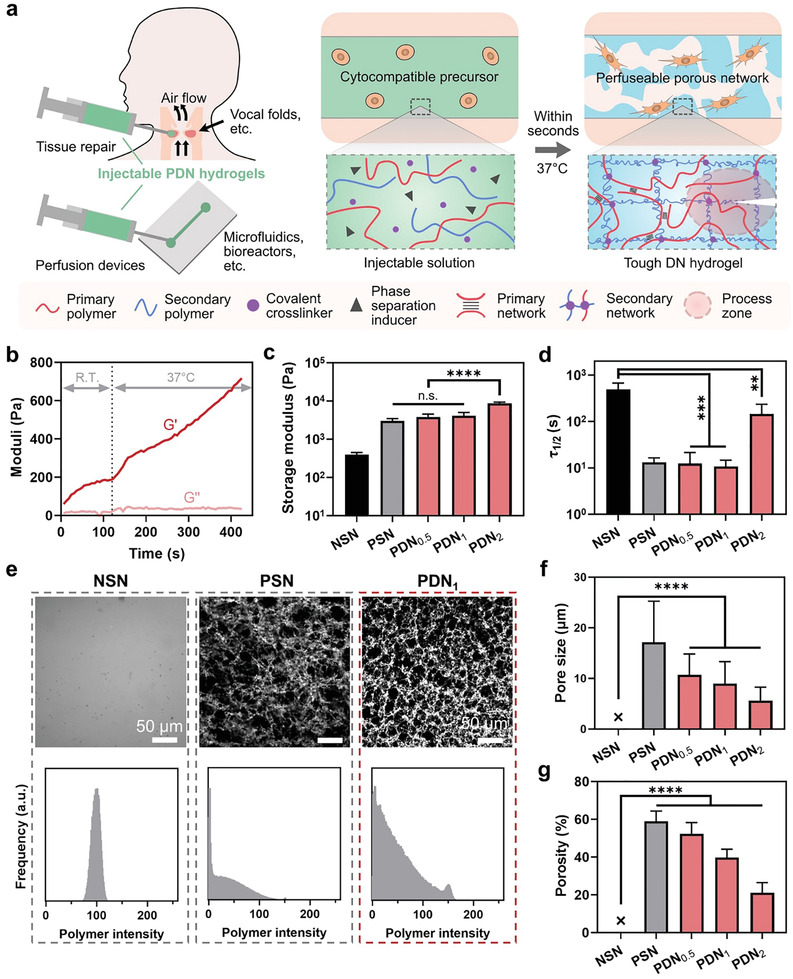
Design and characterization of porous double‐network hydrogels (PDNs). a) PDNs can be used for tissue repair and perfusion devices. They form a tough matrix with micropores in situ in a cytocompatible fashion. b) Thermal gelation kinetics of PDN mixture when the temperature is raised from room temperature (R.T.) to 37 °C. c) Storage moduli and d) half‐life time of stress relaxation (*τ*_1/2_) of NSN, PSN, and PDNs. e) Confocal images (top) and fluorescent signal distribution (bottom) of hydrogels containing FITC‐labeled chitosan and glycol–chitosan, showing the network configurations. f) Pore size and g) porosity of NSN, PSN, and PDNs. Sample size, *N* = 4; ** represents *P* < 0.01, *** *P* < 0.001, **** *P* < 0.0001, n.s. represents *P* ≥ 0.05.

Here, we describe new injectable porous double‐network hydrogels by orchestrating stepwise gelation and phase separation processes. They contain porous double networks, thereby termed PDNs, which differ from previously reported injectable hydrogels that consist of either nanoporous or preformed porous networks. PDNs can form interconnected cell‐sized pores in situ upon injection (**Figure** **1**a). Unlike many porous hydrogels that are weakened by their pores, PDNs are tough and resilient to millions of cycles of mechanical loadings despite the presence of defect‐like pores. They exhibit improved fracture toughness and stretchability compared to nanoporous or porous single‐network counterparts, which are termed NSNs and PSNs, respectively. Both the composition and the gelation method of PDNs are cytocompatible. The highly porous matrices enable rapid medium perfusion and support cell spreading and trafficking. Such perfusable hydrogels can support cell survival in organ‐sized scaffolds with a dimension beyond 60 mm, the largest value reported in the literature to our knowledge. Thanks to the facile injectability, they can be easily delivered through fine needles and incorporated into 3D cell culture perfusion systems with complex mechanical loadings, such as microfluidic chips and bioreactors. We demonstrated that PDNs can withstand over 6000 000 cycles of high‐frequency biomechanical stimulations without rupture. Our method is also generalizable to other material systems. With the unparalleled combination of interconnected pores, toughness, cytocompatibility, and injectability, the described material systems and method may open new opportunities for regenerative medicine and serve as biomimetic in vitro 3D cell culture platforms for a broader range of applications.

## 2 Results and Discussion

### 2.1 Design and Synthesis of PDN

The design of PDN proceeded according to the following criteria: i) cytocompatibility, ii) in situ pore‐forming mechanism, and iii) double‐network framework. To satisfy the first two criteria, we hypothesized that the phase separation of cytocompatible biopolymers at body temperature and physiological pH could both ensure cytocompatibility and generate porous structures in situ. We selected chitosan, a polysaccharide that exhibits phase separation behavior and finds wide uses in biomedical applications,^[^ ^33^, ^34^, ^35^, ^36^, ^37^ ^]^ as an example to test this hypothesis. When the pH of an acidic chitosan solution is raised above its p*K*_a_, 6.5, bicontinuous polymer‐rich and polymer‐poor phases emerge.^[^ ^38^ ^]^ When the polymer‐rich phase is crosslinked, the polymer‐poor phase, comprised mainly of water, results in interconnected open space. This pore‐forming mechanism occurs at physiological conditions, without additional chemical reagents, and is suitable for cell encapsulation and delivery.^[^ ^39^, ^40^ ^]^ Meanwhile, the primary amine groups [NH_3_^+^] of the chitosan deprotonate and are converted to [NH_2_], which can bond with the hydroxyl groups [OH] of the chitosan.^[^ ^41^ ^]^ This self‐crosslinking behavior can stabilize the polymer‐rich phase and thereby reinforce the already‐formed porous structure. Notably, the structure contains a large number of hydrogen bonds and other intermolecular interactions that can be exploited for energy dissipation as the dissipative primary network.^[^ ^33^ ^]^ To satisfy the third criterion, we constructed the secondary network with covalently crosslinked biocompatible polymers. In principle, any polymer that does not affect the phase separation of the dissipative network can be used. Here we used a combination of glycol–chitosan and glyoxal. Glycol–chitosan is a derivative of chitosan with improved solubility at neutral pH. It can be crosslinked by dialdehydes such as glyoxal through a Schiff base reaction to form a secondary network.^[^ ^42^, ^43^ ^]^

To synthesize PDNs, we prepared different concentrations of polymer precursor and gelling agent separately: a PDN precursor comprising chitosan and glycol–chitosan in a weak acetic acid solution; and a gelling agent that contains sodium bicarbonate and glyoxal. Upon mixing of the PDN precursor with the gelling agent, the sodium bicarbonate in the latter raises the pH of the mixture from acidic to neutral and acts as a phase separation inducer to initiate the formation of the dissipative network. Following that, glyoxal chemically crosslinks the glycol–chitosan to form the secondary network. Cells can be incorporated into the mixture before injection. We denoted the resulting hydrogels as PDN*_x_*, where *x* stands for the w/v percentage of glycol–chitosan content. The concentration of chitosan for all the conditions was fixed at 1.5%. NSN hydrogels containing 2% glyoxal‐crosslinked pure glycol–chitosan, and PSN hydrogels containing 1.5% pure chitosan were also synthesized as controls.

### 2.2 Gelation Kinetics, Stiffness, and Stress Relaxation

The gelation of injectable, pore‐forming hydrogels involves three coordinated processes: initial solidification, phase separation, and further crosslinking. The initial solidification should occur in a controlled manner and ensue fast enough to avoid dilution by body fluids. The phase separation and further crosslinking should be separated in time to allow both to proceed independently, leading to interconnected and mechanically stable pores. Our PDN hydrogels meet these design criteria. The precursors and gelling agents reacted and partially crosslinked immediately upon mixing, followed by a gradual stiffening process over time. The initial solidification is reliant on the thermogelling behavior of chitosan (i.e., part of secondary network).^[^ ^40^ ^]^ At room temperature (R.T.), gelation was slow and steady, allowing time for cell encapsulation and injection (Figure 1b). The viscosity at room temperature was also low, which is beneficial for injection with reasonable processing time (Figure S1, Supporting Information). After injection and placement at 37 °C, gelation accelerated, quickly yielding a strengthened hydrogel (Figure 1b). Accompanying the initial solidification, phase separation also took place within seconds. Sequentially, the glycol–chitosan needed around 15 min before starting to crosslink (Figure S2, Supporting Information). The disparate kinetics of the fast phase separation and relatively slow covalent crosslinking ensure the mobility of polymer chains before they are immobilized by chemical bonds, which is essential to the formation of a polymer‐poor phase for the porous structure.

The resulting PDNs showed a favorable viscoelastic response that resembles that of biological tissues. In terms of stiffness, the storage moduli of PDN_0.5_ and PDN_1_ were both ≈3.5 kPa and comparable to that of PSN. Additional covalent network polymers further increased the storage moduli to ≈9 kPa (Figure 1c). The stiffness range spans the range of various biological tissues, such as the vocal folds, lungs, heart, and gastrointestinal tract.^[^ ^9^ ^]^ Young's moduli of PDNs were also significantly higher than NSN and PSN (Figure S3, Supporting Information). Several possibilities could have contributed to this finding. First, the formation of pores concentrates the polymers and crosslinkers at the solid phase, leading to higher crosslinking density and higher moduli. The potential crosslinking between the chitosan and the glycol–chitosan networks by glyoxal could further amplify this effect; second, the polymer concentration of PDN is marginally higher among the three conditions tested, which could contribute to the concentration effect as well. Third, the Young's moduli were measured at small strain ranges, whereas the pores mainly affect the large‐strain behavior (i.e., because of pore collapse). The synergy of these effects strengthens the PDNs despite their high porosity. Meanwhile, all PDNs exhibited a quick stress relaxation behavior. We quantified stress relaxation behavior using the half‐life time, *τ*_1/2_, a matrix to relax to one‐half of its peak value under a constant compressive strain (15%). Stress relaxation time using the stress retention of 1/*e* was also evaluated (Figure S4, Supporting Information). Notably, all the PDNs relaxed within 10^1^–10^2^ s (Figure 1d). This fast stress relaxation response is comparable to that of organs and native extracellular matrix, such as collagen.^[^ ^44^ ^]^ We attribute this behavior to the stress‐induced rupture of hydrogen bonds in the dissipative network, and the fast water migration enabled by the interconnected porous structures, described in a later section. Prompt stress relaxation is beneficial for cell proliferation and migration; it can also help regulate the fate of stem cells.^[^ ^45^ ^]^

### 2.3 Porosity and Permeability

A salient feature of PDNs is their interconnected microporous structure. To characterize the structural properties, we synthesized and visualized the three types of hydrogels (NSN, PSN, and PDN) containing fluorescein isothiocyanate (FITC)‐labeled macromolecules with a confocal microscope at wet state. This process involves no drying or lyophilization treatment. As expected, NSN displayed no detectable pores. The mesh size of NSN and most existing injectable hydrogels is on the order of 10 nm, therefore, well below the resolution limit of the confocal microscope (Figure 1e). In contrast, PSN and all PDNs displayed micrometer‐sized pores resulting from the phase separation of chitosan, which was further confirmed by the fluorescence intensity distribution. A single peak was observed for NSN, indicating a homogeneous network. PSN and PDNs displayed a wide intensity distribution that included areas with low or even no fluorescence. We also verified the porous structures using scanning electron microscopy (SEM) and microcomputed tomography (µCT; Figure S5, Supporting Information). For SEM, the samples were dehydrated with a CO_2_ supercritical dryer to minimize artifacts. For µCT, the samples were scanned in hydrated condition. Both techniques confirmed the presence of an interconnected porous structure within PDNs, concluding the pore‐forming capacity of our hydrogels.

Both the pore size and porosity are tunable by adjusting the concentration of the secondary network polymer, glycol–chitosan. The average pore size varied between 6 and 10 µm, comparable to the size of cells (Figure 1f). The porosity can be tuned over a range of ≈21–54% (Figure 1g). The concentration of glycol–chitosan is inversely proportional to the average pore size and porosity. We attribute this relationship in part to the interplay between the phase separation and the crosslinking of glycol–chitosan. With increasing glycol–chitosan concentration, the crosslinking of glycol–chitosan accelerates, and thus reduces the mobility of the chitosan and the time window for the phase separation. As a consequence, the proportion of the polymer‐poor phase, i.e., pores and porosity, is decreased. The results further underscore the importance of orchestrating the gelation and phase separation processes for desired porous structures.

**Figure 2:**
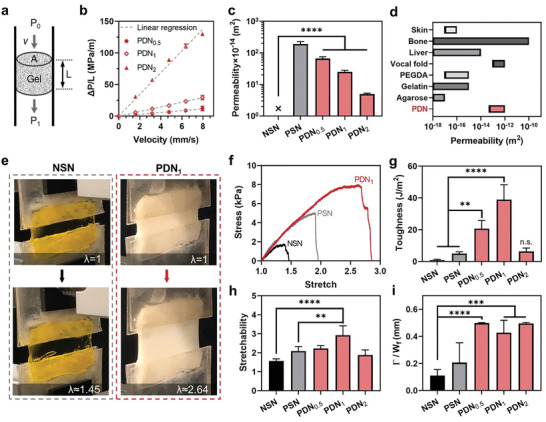
Permeability and toughness of PDNs. a) Schematics of the permeability measurement. The height and cross‐sectional area of the gel are denoted as *L* and *A*, respectively. The flow velocity, up‐ and down‐steam pressures are denoted as *v*, *P*_0_, and *P*_1_. b) Pressure gradient–velocity relations of different gels. c) Permeability of different hydrogels. d) Permeability of different hydrogels and biological tissues (see Table S1 in the Supporting Information). e) Photos showing the stretchability of NSN and PDN_1_. f) Stress–stretch curves of NSN, PSN, and PDN_1_. g) Toughness, h) stretchability, and i) fractocohesive length of NSN, PSN, and PDNs. Sample size, *N* = 4, n.s. represents *P* ≥ 0.05, ** *P* < 0.01, *** *P* < 0.001, **** *P* < 0.0001.

The interconnected porous structures of PDNs enable superior permeability. Permeability governs fluid transport within a hydrogel and mass exchange with the surrounding environment. High permeability supports the survival, activities, and function of cells in deep layers of hydrogels by ensuring adequate nutrient and oxygen delivery. This is especially important when immediate vascularization is lacking. To characterize the permeability, *k*, of PDNs, we perfused cylindrical hydrogel samples with media at various flow rates *Q*, while measuring the pressure drop, Δ*P* = *P*_1_ − *P*_0_, using a pressure transducer (**Figure** **2**a; Figure S6, Supporting Information). Following Darcy's law, we calculated $k\;=\;-\mu \frac{L}{A}\frac{Q}{\Delta P}$, where *μ* is the dynamic viscosity of the media (8.90 × 10^−4^ Pa s for water), and *A* and *L* are the cross‐sectional area and thickness of the hydrogel, respectively. The normalized pressure drop (Δ*P*/*L*) across the PDN sample was linearly proportional to flow velocity, confirming an ideal porous material flow resistivity behavior (Figure 2b). The permeability of PDNs was on the order of 10^–14^–10^–12^ m^2^ (Figure 2c). In contrast, it was not possible to perfuse media through NSN without fracturing the hydrogel due to its low permeability. We also compared the permeability results with values for commonly used hydrogels and biological tissues (Figure 2d). PDNs exhibited at least 2–4 orders of magnitude greater permeability than most existing hydrogels. The measured permeability demonstrates that the PDNs contain highly interconnected porous structure, enabling rapid convection of transport fluid within the matrix.

### 2.4 Toughness and Pore Insensitivity

Despite the highly porous structure, PDNs are mechanically tough and insensitive to pores. We measured the toughness with pure shear tests (Figure 2e). The area under the stress–stretch curve before a critical stretch, which was measured with a notched specimen, is the critical energy release rate to drive crack propagation (Figure 2f). The toughness values of NSN and PSN were ≈1 and ≈5 J m^–2^, respectively. These values agree with past reports on the toughness of single‐network hydrogels (1–10 J m^–2^).^[^ ^46^ ^]^ In comparison, PDN_0.5_ and PDN_1_ exhibited fracture toughness values of 20 and 39 J m^–2^, respectively, corresponding to 20‐ and 40‐fold increases compared to NSN (Figure 2g). The stretchability of PDN_1_ was also twice higher than that of NSN (Figure 2h). The enhanced toughness and stretchability of PDNs are attributed to the double‐network configuration. Presumably, the physical crosslinks of chitosan break to dissipate energy under strain, while the covalent crosslinks of glycol–chitosan retain structural integrity. Notably, the toughening performance from the phase separation of chitosan was more significant compared to commonly used dissipative network, such as calcium crosslinked alginate. We prepared nanoporous double‐network (NDN) hydrogel by replacing chitosan with calcium alginate within PDN. Although NDN improved the toughness by fivefold compared to NSN, the achieved toughness was still one order of magnitude lower than that of PDN_1_ (Figure S7, Supporting Information). To evaluate the pore sensitivity of the hydrogels, we compared their fractocohesive lengths, characteristic crack lengths below which a material is insensitive to its presence. Unlike single‐network hydrogels, for which pores act as defects, our PDNs demonstrated a fractocohesive length of up to 0.5 mm (Figure 2i). Owing to this high flaw‐insensitive threshold, the pores within the PDNs did not degrade their toughness or act as defects. A detailed summary of toughness, permeability, pore size, porosity, and stress relaxation data is shown in Table S1 (Supporting Information).

### 2.5 Swelling and Degradability

As swelling affects the mechanical and physical robustness, the swelling profile of PDNs was evaluated next. Due to difficulties in accurately measuring the weight of hydrated porous materials, we quantified the swelling by monitoring the dimensional change of PDNs upon immersion in phosphate‐buffered saline (PBS). PDNs maintained their original sizes with less than 10% size change, while swelling greater than 30% was observed in NSN over a 7 day period (Figure S8, Supporting Information). Good physical stability in a liquid environment helps hydrogels maintain their shape, which is important in ensuring that surrounding tissues are not subjected to undue compressive stresses. PDNs are also biodegradable by enzymes. They showed a slow degradation profile over 28 days when exposed to lysozyme at the physiological level (Figure S9, Supporting Information). Such a degradation rate is helpful in supporting the growth of encapsulated cells while they secrete their own matrix to form new tissue.

### 2.6 Cytocompatibility

**Figure 3:**
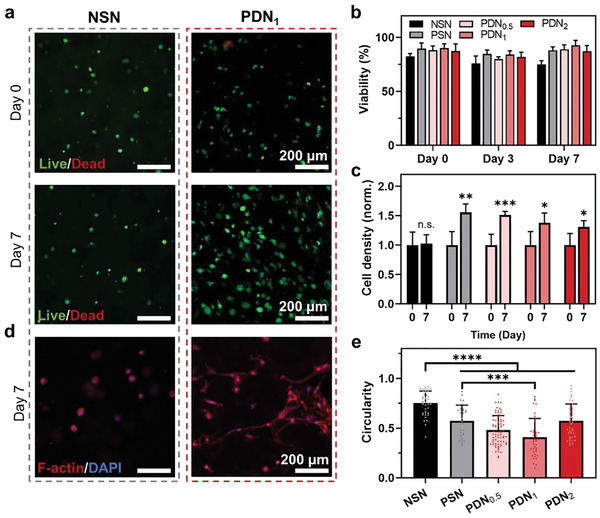
Biological properties of PDNs. a) Confocal images of live/dead cells cultured within hydrogels on days 0 and 7. b) Cell viability over time. c) Normalized cell density over time. d) Confocal images showing the morphology of cells cultured within hydrogels on days 0 and 7. e) Circularity of cells cultured within different hydrogels on day 7. *** represents *P* < 0.001, **** represents *P* < 0.0001. Sample size, *N* = 4.

Injectable hydrogels for cell encapsulation and delivery must be cytocompatible and supportive of cell growth. These biological characteristics of PDNs were evaluated with human vocal fold fibroblasts (hVFFs), one of the main cell types found in mechanically dynamic vocal fold tissues.^[^ ^47^ ^]^ The cells were encapsulated within the hydrogels during a 7 day culture. LIVE/DEAD assays showed that all the NSN, PSN, and PDNs are cytocompatible. The cell viability for PDNs exceeded 85% in all cases and was consistently higher than that of NSN (**Figure** **3**a,b; Figure S10, Supporting Information). Hydrogels used here were not fluorescently labeled and thus not visualized. Substantial hVFFs’ proliferation further confirmed that PDNs provided a cell‐friendly 3D environment (Figure 3c). In contrast, cells cannot proliferate in NSN, likely due to the nanoporous matrix imposing excessive mechanical constraints that restricted cellular activities. A similar conclusion was drawn from assessments of cell morphology. The hVFFs elongated within the 3D porous matrices of PDNs, while those cultured in NSN maintained spherical shapes (Figure 3d; Figure S11, Supporting Information). Figure 3e shows a substantial difference in cell circularity between the nanoporous and porous gels, supporting the importance of porous structure in promoting cell spreading (Figure 3e). Cells were also found to penetrate into PDN_1_ matrix within a 2 day culture period under a chemoattractant gradient but not NSN (Figure S12, Supporting Information). The result demonstrates the function of pores in facilitating cell recruitment and migration. Considering its excellent mechanical, structural, and biological properties, PDN_1_ was chosen for the subsequent investigations, unless otherwise specified.

### 2.7 Use of PDN in Microfluidics

**Figure 4:**
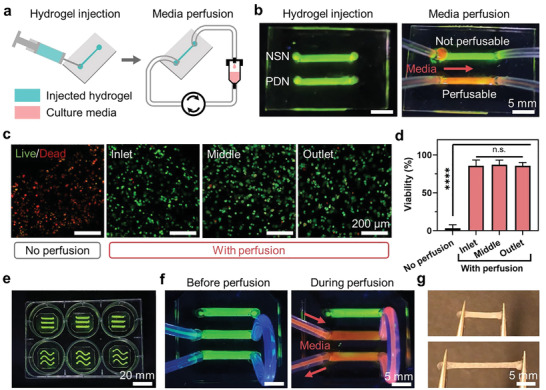
Injectable PDN for microfluidics. a) Schematics showing the delivery of PDNs to microfluidic channels through injection. b) Perfusion of media through injected hydrogels. c) Confocal images showing live/dead cells within PDNs with and without perfusion after 24 h. d) Cell viability. e) PDN‐enabled microfluidic setup within a standard 6‐well plate. f) Different channels can be connected and perfused simultaneously. g) Injected PDNs can be taken out from the microfluidic channels and handled easily without rupture. **** represents *P* < 0.0001, n.s. represents *P* ≥ 0.05. Sample size, *N* = 3.

We first explored the use of PDN in a miniaturized perfusable 3D culture device, where PDN was injected into a microfluidic channel (**Figure** **4**a). Their injectability eliminates the need for high‐precision prefabrication of tight‐fitting inserts for microfluidic devices. To visualize the function of the device, we prepared FITC‐labeled hydrogels (green) and added rhodamine dyes into PBS to simulate perfusion media (red). Figure 4b shows the direct media perfusion through the PDN matrix, thanks to its interconnected porous structures and high permeability. No channel blockage or media leakage was observed. By examining the distribution of the fluorescent signals, we confirmed that media perfused through the entire channel. In contrast, NSN blocked the microfluidic channels and prevented media flow.

To demonstrate the cell culture with PDN in microfluidics, we cultured hVFFs in the PDN‐laden devices. The hVFFs were mixed into the hydrogel precursors and injected into the device's channels. The cell‐laden device was then perfused with cell culture media for 24 h. A standard culture condition without perfusion was included as control. Viability assays confirmed perfect cell viability throughout the PDN channel, including the inlet, the middle, and the outlet (Figure 4c,d). In contrast, most cells were dead in the control due to a lack of oxygen and nutrients. The setup is also readily usable in well plates, similar to those used in microfluidic devices (Figure 4e). Notably, the high permeability and low flow resistance of PDNs allow the perfusion of multiple channels connected in series with a single syringe input (Figure 4f). This enables a modular design for the coculture of multiple cells in different compartments. Owing to their mechanical toughness, the injected PDNs can be easily harvested and manipulated after their maturation (Figure 4g). They can be sectioned to perform multiple assays in parallel, such as different immunochemistry staining tasks. PDN shows promise in simplifying the design and operation of 3D cell culture microfluidics.

### 2.8 Performance under Extreme Biomechanical Stimulations

**Figure 5:**
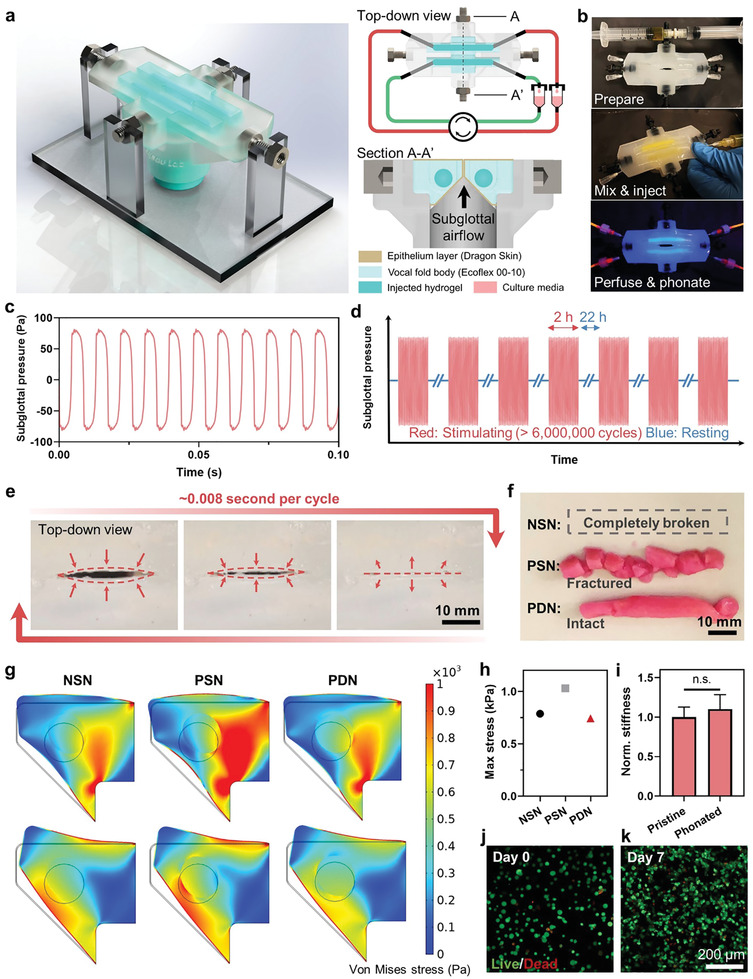
Stability of PDN under highly dynamic and cyclic biomechanical stimulations. a) Schematics showing the design and setup of a phonomimetic bioreactor. b) PDN being injected into the bioreactor. c) Close‐up view of subglottal pressure profiles applied on the injected hydrogels. d) Injected hydrogels experience more than 6 million high‐frequency cyclic mechanical stimulations. e) The movement of bioreactor lips within one cycle from the top‐down view. f) Digital photos showing the morphologies of injected hydrogels after mechanical stimulations. g) Finite‐element simulations showing the stress distribution within the solid phases of the bioreactor and the injected hydrogels. Black contours indicate the un‐deformed shape, and the inner circle refers to the hydrogel. The two rows represent the two positions during one phonation cycle where the hydrogel experiences the highest stresses. h) Maximum stress experienced by different hydrogels. i) Stiffness of the pristine PDN and the PDN after being stimulated in the bioreactor for 7 days. j) Confocal images showing the live/dead cells cultured within injected PDNs after phonated for 7 days. k) Viability data. n.s. represents *P* ≥ 0.05. Sample size, *N* = 3 or 4.

To test the resilience of PDN under complex physiological conditions, we used a phonomimetic perfusable bioreactor to simulate biomechanical stimulations of the vocal folds (Figure S13, Supporting Information).^[^ ^48^ ^]^ **Figure** **5**a illustrates the structure of the bioreactor, which contains a pair of elastomer‐based vocal fold bodies covered by a thin outer layer, representing the lamina propria and the epithelium of the real tissue. Each vocal fold body contains a cavity where the PDN can be easily injected, perfused, and be subjected to phonation stresses thereafter. Medium is perfused through hypodermic needles inserted through the elastomer to reach the cavity and ensure a hemetic seal (Figure 5b). Phonation is achieved with controlled airflow across the subglottal area that induces self‐oscillation of the vocal fold bodies (Figure 5a, Sections A–A′). The phonation frequency and subglottal pressure were controlled to within physiologically relevant range (Figure 5c). In particular, the frequency was kept at ≈120 Hz, similar to the fundamental frequency of humans when voicing. The injected hydrogels were phonated for 2 h/day for 7 days, inducing a total of over 6000 000 cycles of vibrations (Figure 5d). The lips of the bioreactor close, collide, and open during each phonation cycle that lasts 0.008 s (Figure 5e; Movie S1, Supporting Information). After the completion of biomimetic stimulations, we harvested the hydrogels from the bioreactor and found that PDN withstood the extreme biomechanical environment and maintained their integrity (Figure 5f). The porous structure was also found similar to the pristine state after cyclic loading under perfusion (Figure S14, Supporting Information). In contrast, NSN disintegrated into small particles that were washed away by the perfusion media, and PSN fractured into multiple disjoint chunks.

To further reveal the mechanical environment in the bioreactor, we conducted finite‐element analysis to probe the mechanical loading applied onto the hydrogels (Figure 5g; Figure S15 and Movie S2, Supporting Information). Both elastomers and hydrogels were treated as hyperelastic materials with the Ogden model and conjugated with damping. The PDN model has the lowest maximum von Mises stress and the most homogeneous stress distribution among the three conditions, due to its excellent energy dissipation under stress (Figure 5h). We also verified experimentally that the stiffness of PDN was not affected by the cyclic mechanical stimulations (Figure 5i). Despite the complex loadings, PDN was found to support cellular viability and proliferation for the encapsulated cells throughout the 7 day culture period (Figure 5j,k). Cell culture media were able to penetrate the entire 6 cm thick scaffold thanks to its exceptional permeability. To our knowledge, this is the first material reported to support cell viability in a centimeter‐scale avascular construct.

PDN also shows great translational potential. We found that encapsulated hVFFs secreted more collagen content under dynamic stimulations compared to cells that cultured statically, indicating that the stability of PDN could help activate encapsulated cells to produce a functionalized tissue (Figure S16, Supporting Information). In addition, we demonstrated that PDNs can be easily injected into animals subcutaneously to form a porous hydrogel in situ without leakage (Figure S17a, Supporting Information). The collective evidence supports the hypothesis that PDNs can be used to repair mechanically active tissues such as vocal folds after lesion removal and calls for future investigations (Figure S17b, Supporting Information). It is also valuable to explore and expand the use of PDN for other cell systems such as stem cells and organoids in future studies.

### 2.9 Generalizability

We next discussed how our design strategy of PDN hydrogels could be extended to other material systems. Since the phase separation of chitosan occurs under mild conditions, our strategy could be compatible with other materials and polymer crosslinking strategies, including free‐radical polymerization. As an example, we replaced glycol–chitosan with gelatin, another widely used biopolymer, for the secondary network. We prepared a gelatin‐based PDN precursor by dissolving gelatin and chitosan in an acetic acid solution while keeping the same gelling condition. In the new material system, the gelatin can also be crosslinked by glyoxal and the chitosan still ensues phase separation. As expected, the resulting gelatin‐based PDN showed an interconnected porous structure (Figure S18, Supporting Information). The toughness of the resulting PDN was also higher compared to that of pure gelatin hydrogel. Chitosan could be potentially substituted with other thermogelling polymers such as poly(*N*‐isopropylacrylamide).^[^ ^49^ ^]^ It is worth noting that although PDNs are relatively weak in comparison with traditional tough hydrogels, they provide significantly improved toughness and fatigue resistance compared to existing injectable hydrogels and perform stably under the most extreme conditions present in the human body. Given the diversity of polymer systems, the present work may well give rise to a new class of injectable microporous hydrogels that are tough, perfusable, and easy to use.

## 3 Conclusion

We described a methodology to endow injectable hydrogels with superior permeability, toughness, and cytocompatibility simultaneously. The toughness and stretchability of PDNs overperformed those of traditional injectable materials, while the in situ forming porous structures enabled direct media perfusion throughout the bulk matrices. The hydrogels were also favorable to cell growth, spreading, and proliferation. They maintained their structural integrity under highly dynamic cyclic biomechanical loadings while supporting cell viability and functions. Their great potentials were demonstrated for the use in cell‐culture perfusion microfluidics and vocal fold mimetic perfusion bioreactors. Thanks to an unprecedented combination of mechanical, structural, and biological properties, the proposed material and technology are expected to impact broadly the repair and regeneration of mechanically dynamic tissues and benefit the development of drug delivery, microfluidics, cell culture, and disease modeling.

## 4 Experimental Section

### Hydrogel Synthesis

Chemicals used in the current work were purchased from Sigma–Aldrich and used without further purification unless stated otherwise. Chitosan (DDA: 95%, medium and high molecular weights) was purchased from Xi'an Lyphar Biotech. Pure chitosan (PC) powder was dissolved and stirred in 0.2 m acetic acid to form a homogeneous chitosan solution. Different concentrations of glycol‐chitosan (denoted by GC, G7753) were added to the chitosan solution to form PDN precursors. To prepare the gelling agents, a phosphate solution (PS) was first prepared by mixing 0.1 m sodium phosphate dibasic (Na_2_HPO_4_, S7907) and 0.1 m sodium phosphate monobasic (NaH_2_PO_4_, S8282) with a volume ratio of 50:3. The gelling solutions were then completed by adding glyoxal (50649) and sodium bicarbonate (denoted by SC, S233‐500, Fisher Scientific) into the phosphate solution. A hydrogel precursor and its associated gelling agent could be mixed at a volume ratio of 3:2 using a syringe connector to yield hydrogels. Materials for synthesizing control groups included alginate (ULV‐L3G, KIMICA Corporation), gelatin type A (G2500), and CaSO_4_ (C3771). The detailed ingredients for each formulation are listed in **Table** **1**.

**Table 1:** The detailed ingredients for each formulation

Hydrogel	Hydrogel precursor	Gelling agent
NSN	3.33% GC in PBS	0.0124% glyoxal in PBS
PSN	2.5% PC in 0.2 m acetic acid	0.445 m SC in PS
PDN_0.5_	0.84% GC + 2.5% PC in 0.2 m acetic acid	0.445 m SC + 0.0031% glyoxal in PS
PDN_1_	1.67% GC + 2.5% PC in 0.2 m acetic acid	0.445 m SC + 0.0062% glyoxal in PS
PDN_2_	3.34% GC + 2.5% PC in 0.2 m acetic acid	0.445 m SC + 0.0124% glyoxal in PS
Pure gelatin	4.17% gelatin in PBS	0.0155% glyoxal in PBS
Gelatin–PDN	1.67% gelatin + 2.5% PC in 0.2 m acetic acid	0.445 m SC + 0.0062% glyoxal in PS
NDN	1.67% GC + 2.5% alginate in water	0.1 m CaSO_4_+ 0.0062% glyoxal in water

### Mechanical Characterizations

Gelation kinetics and frequency sweeps were measured using a torsional rheometer (HDR‐2, TA Instruments) with parallel plates (upper plate diameter of 20 mm). The shear moduli of hydrogels were obtained from isothermal time sweeps at a frequency of 0.1 Hz and 0.1% strain at 37 °C for 2 h. Frequency sweeps ranging from 0.01 to 100 Hz at 0.1% strain and 37 °C followed to determine the damping ratios. Relaxation moduli were obtained by holding a step compressive strain of 15% using an Instron machine (Model 5965, 10 N load cell) and measuring the compressive stress–time profiles.

The fracture energy or toughness of hydrogels was determined using pure shear tests. One pair of samples was used for each data point. One sample was un‐notched, and the other sample was notched. In their un‐deformed state, each sample had a width of *W* = 40 mm and a thickness of *T* = 1.5 mm. The distance between the two polyester clamps was *H* = 5 mm. The un‐notched sample was pulled by an Instron machine with a 10 N load cell at a strain rate of 2 min^–1^ to measure the stress–stretch (*S*–*λ*) curve. For the notched sample, a notch length of ≈10 mm was introduced using a razor blade. The notched sample was pulled until rupture to obtain the critical stretch (*λ*_c_). The fracture energy was calculated using the *S*–*λ* curve from the un‐notched sample: $\Gamma \;=\;H\int_{1}^{\lambda_{c}}sd\lambda$.

### Structural Characterizations

The polymer network was imaged using a confocal microscope (LSM 710, Zeiss). Both chitosan and glycol–chitosan were conjugated with FITC fluorescent labels according to published protocols.^[^ ^50^, ^51^ ^]^ Samples were prepared by mixing fluorescent‐labeled polymer solutions and crosslinkers in a vial and transferring ≈150 µL into a 35 mm Petri dish with a coverslip bottom (P35G‐0‐10‐C, MatTek). Hydrogels were immersed under PBS and imaged as prepared. The polymer network was imaged with 10× and 20× objective lenses.

Macro‐ and microscopic pores were also imaged using a field‐emission scanning electron microscope (F50, FEI) under various magnifications. Before SEM imaging, all samples were immersed inside 30%, 50%, 70%, 80%, 90%, and 100% ethanol in sequence for dehydration. Ethanol inside the hydrogels was removed using a CO_2_ supercritical point dryer (CPD030, Leica) to preserve the original pore size. The dehydrated samples were coated 4 nm Pt using a high‐resolution sputter coater (ACE600, Leica) to increase surface conductivity.

Imaging with µCT was performed using a SkyScanner 1172 (Bruker) through a 360° flat‐field corrected scan at 30 kV and 112 µA, with a rotational step size of 0.45°, a cross‐sectional pixel size of 6.5 µm, and no filter. The samples were prepared and incubated at 37 °C for 24 h. The volumetric reconstruction (NRecon, Micro Photonics) was performed with a beam hardening correction of 40%, a ring artifact correction of 4, and an auto‐misalignment correction. The 2D and 3D analyses were carried out using Dragonfly software and a grayscale intensity range from 50 to 70 (8 bit images) to remove background noise.

### Permeability Measurement

A customized t‐shaped adaptor was 3D‐printed to enable a controlled application of pressure to force the test fluid through hydrogels. Before testing, a hydrogel was first cured inside the hydrogel container at 37 °C. The container was then enclosed by slotting it into the main body and screwing on the retaining cap. The pressure sensor was then connected, and the modified syringe connectors were opened. A liquid‐loaded syringe was then connected to the perpendicular port, and the adaptor was slowly filled while ensuring all the air escapes. The air outlet was then sealed before the test began. During the test, the syringe pump was set to advance at a fixed rate and the pressure was measured. The fluid that passes through the hydrogel was collected and measured using the stopwatch and bucket method. The measured pressure and volume were used to calculate the permeability of the gel according to Darcy's law $q=-\frac{k}{\mu L}P$.^[^ ^52^ ^]^

### Cell Culture

Immortalized hVFFs were cultured in Dulbecco's modified Eagle medium (DMEM, Corning) containing sodium pyruvate and supplemented with 10% fetal bovine serum, 1% penicillin/streptomycin, and 1% MEM nonessential amino acids. Cells were incubated at 37 °C, in a 5% CO_2_ humidified atmosphere. The media were changed every 3 days for 2D cultures. Cells were disassociated using 0.25% trypsin‐ethylenediaminetetraacetic acid (EDTA) when the cell confluency reached 70%.

### Cytocompatibility

To evaluate the cytocompatibility of hydrogels, hVFFs were suspended in hydrogel mixtures immediately after the precursors with gelling agents were mixed. The final cellular concentration was 1 million mL^−1^. The mixtures were then injected into Petri dishes to form hydrogels. Complete DMEM with 10% fetal bovine serum (FBS) was used as cell culture medium and changed every day. hVFFs were stained by a LIVE/DEAD viability kit (L3224, Invitrogen) inside 3D matrices on days 0, 3, and 7. Imaging of fixed hVFFs was conducted using a confocal laser scanning microscope (LSM710, Zeiss, Germany). Live cells were shown in green fluorescence and dead cells were shown in red.

### Cell Penetration

To evaluate the cell penetration into the hydrogels, hVFFs cultured in 2D flasks were first starved in serum‐free DMEM for 6 h. Cells were then detached and suspended in serum‐free DMEM at a concentration of 50 000 cells mL^−1^. 200 µL of cell‐free hydrogels was coated on cell culture inserts (08‐771‐10, Fisher Scientific) to evenly cover the permeable membrane of 0.45 µm pore size. Serum‐free cell suspension (0.8 mL) was added on top of each hydrogel. The cell inserts were then placed into a 12‐well plate. Serum‐rich DMEM containing 10% FBS was then added to the wells and outside of cell culture inserts to form a chemoattractant gradient across the permeable membrane. Cells were cultured for 2 days before being counterstained with 4′,6‐diamidino‐2‐phenylindole (DAPI, D1306, Invitrogen) using a 1:5000 dilution for 5 min, followed by rinsing twice with PBS. *Z*‐stack imaging of cell penetration into the hydrogels was conducted using a confocal laser scanning microscope (LSM800, Zeiss, Germany).

### Immuno‐Histochemistry

Hydrogels were first washed with prewarmed PBS twice and then fixed in 3.7% formaldehyde solution for 15 min. The fixed samples were washed with PBS again twice and permeabilized with 0.1% Triton X‐100 in PBS for 5 min. The samples were blocked in 1% bovine serum albumin (BSA, A1595) for 1 h. To conduct F‐actin staining, 10 µL of Alexa Fluor 633 Phalloidin (A22284, Invitrogen) was diluted into 200 µL PBS containing 1% BSA. The samples were incubated inside the staining solution at room temperature for 30 min followed by three times PBS wash. To conduct collagen staining, rabbit polyclonal antibody of collagen‐I (1:200, ab34710, Abcam) was added to PBS containing 1% BSA. The samples were incubated inside the collagen staining solution at room temperature for 30 min followed by three times PBS wash. The samples were blocked again in goat serum and then incubated for 1 h with the Alexa Fluor 488 goat antirabbit immunoglobulin G (IgG) secondary antibody (1:1000, A11034, Invitrogen) followed by three times PBS wash. The nuclei were counterstained with DAPI using a 1:5000 dilution for 5 min, followed by rinsing twice with PBS.

### Swelling and Biodegradation

The swelling ratios were determined by immersing the hydrogel disks (10 mm in diameter, 1.5 mm in thickness) in PBS (pH = 7.4) at 37 °C with gentle mechanical stimulation (75 rpm). The diameters of the disks were measured using a caliper at predetermined time intervals using a pipette. The swelling ratio was calculated by dividing the measured diameter size by the initial value. For biodegradation assays, all hydrogel samples were prepared with the same volume (500 µL). The average dry weight of the pristine hydrogels was used as the weight at day 0. After that, an enzyme solution consisting of 13 µg mL^−1^ lysozyme (100831, MP Biomedicals) in PBS was added to the gels. The samples were incubated at 37 °C with gentle mechanical stimulation over 28 days. The enzyme solution was changed every other day. At predetermined time intervals, the enzyme solution was removed. The samples were then washed three times for 5 min with PBS. The samples were then lyophilized and the remaining polymer dry weight was measured. The remaining ratio of the polymer was calculated by dividing the dry weight of the remaining polymer by the dry weight of the initial gels.

### Microfluidic Devices

The body of the microfluidic devices was fabricated using soft lithography.^[^ ^53^ ^]^ In brief, a negative mold was created by printing a Pluronic F‐127 ink (37 wt% in deionized water, P2443) inside a Petri dish into predefined patterns with a bioprinter (BioAssemblyBot, Advanced Solutions). Polydimethylsiloxane (PDMS, SYLGARD 184, Dow) was prepared by mixing the base to cure at a weight ratio of 10:1. PDMS was degassed and poured into the Petri dish to cover the printed constructs. After curing at 60 °C overnight, the cured PDMS was taken out of the mold. Pluronic F‐127 was removed by washing in cold water. The surfaces of the PDMS body and glass slide were treated with oxygen plasma before bonding to form the complete device. A 2 mm biopsy punch was used to create openings for the inlets and outlets. Devices were repeatedly sterilized with 70% ethanol before washing with PBS. Hydrogels were injected to fill the microfluidic channels. The devices were incubated at 37 °C for 30 min before flow perfusion.

### Bioreactor

The detailed bioreactor fabrication steps can be found in Figure S13 (Supporting Information). Sterile needles (305198, BD Medical) were first inserted from the two sides of the bioreactor body until reaching the empty hydrogel chamber. Hydrogel precursors and their associated gelling agent were quickly mixed, followed by mixing in a cell suspension to reach a final concentration of 2 million mL^−1^. The cell‐laden hydrogel precursors were then injected through preinserted needles to fill the chambers. The bioreactor was then placed inside an incubator. Hydrogels were left to crosslink for 2 h before cell culture media were perfused. The average perfusion flow rate was 5 µL min^−1^. The bioreactor was phonated for 2 h per day over 7 days. Dynamic subglottal and supraglottal pressures were monitored using two pressure transducers (130D20, PCB Piezotronics) placed 10 cm below and above the bioreactor lips, respectively. The microphone was connected to a conditioning amplifier (Brüel & Kjær) that connected to a data acquisition system (National Instruments). Digital readouts for flow and pressure were displayed on a PR 4000F (MKS Instruments). Hydrogels were harvested after predetermined time points for various assays.

### Numerical Simulations

COMSOL Multiphysics (Stockholm, Sweden) was employed to simulate the phonation in the vocal fold bioreactor. A 2D fully coupled fluid‐structure interaction (FSI) model was developed using the unsteady Navier–Stokes equations for the fluid domain. The solid domain consisted of three parts representing the lamina propria (hydrogels), vocalis muscle (Ecoflex 00–10), and a thin epithelium layer (Dragon Skin). The hyperelastic Ogden material model was used for the solid domain. The strain energy density for the Ogden model is given by

(1) $$\psi_{D}=\frac{\mu }{\alpha }\;\left(\lambda_{1}^{\alpha }+\lambda_{2}^{\alpha }+\lambda_{3}^{\alpha }-3\right)$$

where *ψ*_D_ is the strain energy density, *μ* and *α* are the fitting coefficients, and *λ*_*i*_ is the *i*th principal stretch. The nominal stress *S* for a pure shear test is given by

(2) $$S\;=\;\mu \left(\lambda^{\alpha -1}-\lambda^{-\left(\alpha +1\right)}\right)$$

The Ogden parameters for the hydrogels were determined by fitting Equation (2) into the loading paths in the pure shear test results of the hydrogels. The Ogden parameters for the Ecoflex 00–10 and Dragon Skin were extracted from the previous measurement.^[^ ^48^ ^]^

Rayleigh damping model was used for the hydrogels. A dynamic system can be described by

(3) $$\left[M\right]\frac{d^{2}x}{dt^{2}}+\left[C\right]\frac{dx}{dt}+\left[K\right]x\;=F_{\mathrm{static}}\;+F_{\mathrm{dynamic}}$$

where [*M*] is the mass matrix, [*C*] is the damping matrix, [*K*] is the stiffness matrix, *x* is the displacement as a function of time, and *F*_static_ and *F*_dynamic_ are the static and dynamic loads, respectively. The system damping matrix is defined by

(4) $$\left[C\right]=\;\delta \left[M\right]+\beta \left[K\right]$$

where *δ* and *β* are the mass and stiffness proportional Rayleigh damping coefficients, respectively. The Rayleigh damping coefficients were determined by the different damping ratios *ξ* at different response frequencies *ω* in rad s^−1^ according to

(5) $$\xi \;=\frac{1}{2}\;\left(\frac{\delta }{\omega }+\beta \omega \right)$$

The damping ratios, $\xi \;=\frac{1}{2}\;\frac{G^{\prime \prime }}{G^{\prime }}$, were measured from frequency sweep between 0.1 and 100 Hz using a rheometer, where *G*′ and *G*″ are the storage and loss moduli, respectively. Damping in the two other solid bodies was modeled using the isotropic loss factor to account for the intrinsic damping properties of the materials. For the fluid domain, the no‐slip boundary condition was applied on the surface of the elastomers. The inlet airflow was defined as an incompressible fully developed laminar flow at room temperature.

To reduce the computational cost, a symmetric one half‐body of the M5 vocal fold model was designed from an existing canonical model^[^ ^54^ ^]^ for glottal airflow simulation. Dynamic free triangular fine meshes were used to allow for the FSI modeling. An implicit time‐dependent fluid solver with a step size of 0.001 s was used in conjunction with a physically controlled tolerance. The stress distribution within the elastomers and the hydrogels was obtained from the simulations. Tables S2 and S3 (Supporting Information) present the simulation parameters and the material properties, respectively.

### Statistical Analysis

A sample size of *N* ≥ 3 was used for all experiments. Data were shown as mean ± standard deviation (SD). Statistical analysis was performed using one‐way analysis of variance (ANOVA) and posthoc Tukey tests for multiple comparisons or Student's *t*‐tests for comparison between two groups (Prism 9, GraphPad Inc.). *P*‐values of <0.05 were considered statistically significant.

## Conflict of Interest

G.B., J.L., L.M., and S.T. are inventors of a patent application (US patent 63/278,288) that covers the design and materials of PDN.

## Author Contributions

S.T. and G.B. contributed equally to this work. G.B., J.L., and L.M. conceived the idea, designed the study, and supervised the project. G.B. and S.T. developed the materials and method for the hydrogels. Z.H. designed and optimized the bioreactor system. S.T., G.B., Z.H., S.M., and H.R. carried out the experiments. H.R., G.B., and S.T. conducted numerical simulations. G.B., S.T., J.L., and L.M. analyzed the results and wrote the manuscript with inputs from all authors.

## Supporting information

Supporting Information

Supplemental Movie 1

Supplemental Movie 2

## Data Availability

The data that support the findings of this study are available from the corresponding author upon reasonable request.
