# Supplementary material for: Injectable, Pore‐Forming, Perfusable Double‐Network Hydrogels Resilient to Extreme Biomechanical Stimulations
Source: Adv Sci (Weinh). 2021 Nov 22;9(2):2102627. doi: 10.1002/advs.202102627 (PMC8805581; doi:10.1002/advs.202102627)
Supplement: Supplementary file 1 — Supporting Information [file ADVS-9-2102627-s003.pdf]

ADVANCED SCIENCE
Open Access

Supporting Information

for *Adv. Sci.*, DOI: 10.1002/advs.202102627

Injectable, pore-forming, perfusable double-network hydrogels resilient to extreme biomechanical stimulations

Sareh Taheri, Guangyu Bao, Zixin He, Sepideh Mohammadi, Hossein Ravanbakhsh, Larry Lessard, Jianyu Li*, and Luc Mongeau**

Supporting Information

Injectable, pore-forming, perfusable double-network hydrogels resilient to extreme biomechanical stimulations

Sareh Taheri, Guangyu Bao, Zixin He, Sepideh Mohammadi, Hossein Ravanbakhsh, Larry Lessard, Jianyu Li*, and Luc Mongeau**

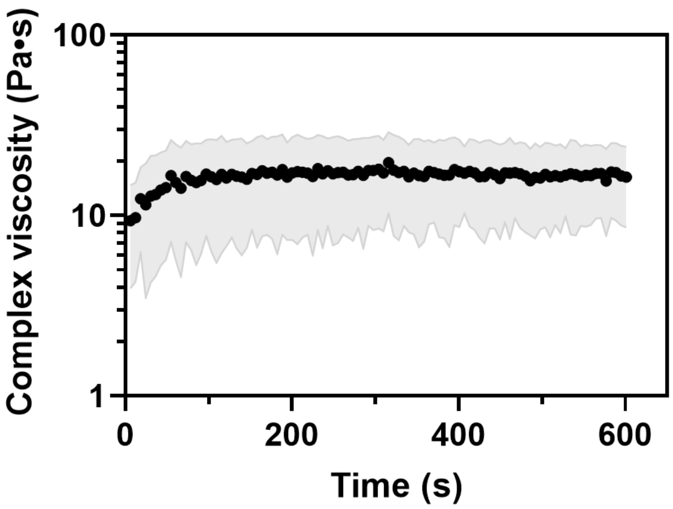

Figure S1. Viscosity of PDN_1 as a function of time after mixing at room temperature. Black dots denote the mean value, and the grey area denotes the standard deviation. Sample size, *N* = 3.

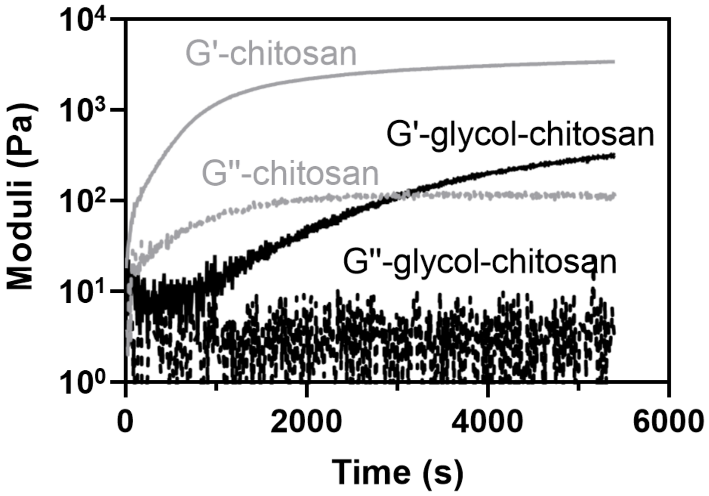

Figure S2. Gelation kinetics of chitosan and glycol-chitosan. The primary network (chitosan) crosslinks within seconds, while the secondary network (glycol-chitosan) did not occur within 15 mins. The disparate kinetics provides a sufficient time window for pore formation.

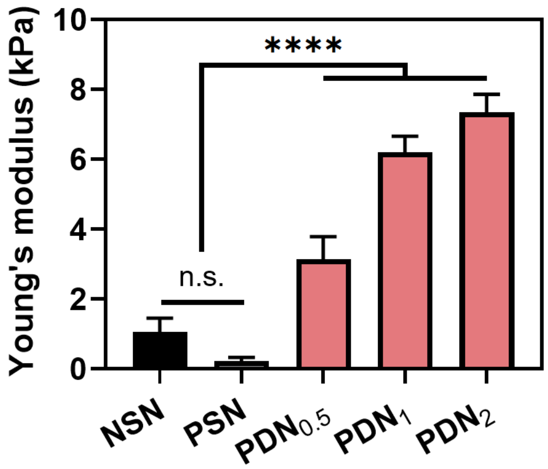

Figure S3. Young's moduli of NSN, PSN, and PDNs calculated from compression tests. Sample size, $N = 4$. **** $P < 0.0001$, n.s. means $P \geq 0.05$.

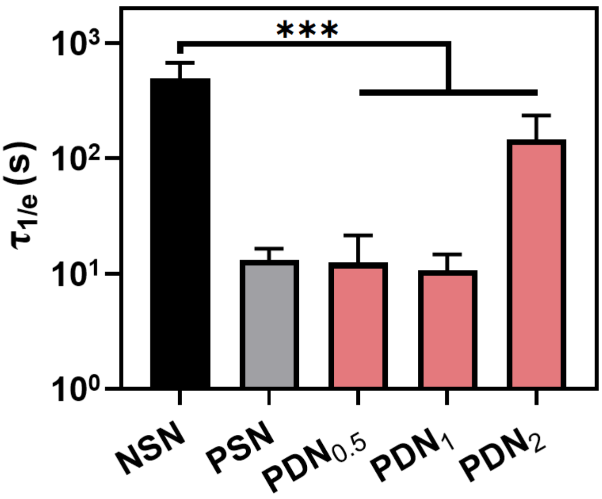

Figure S4. Stress relaxation time of different hydrogels. Stress relaxation was evaluated by the stress retention of 1/e.

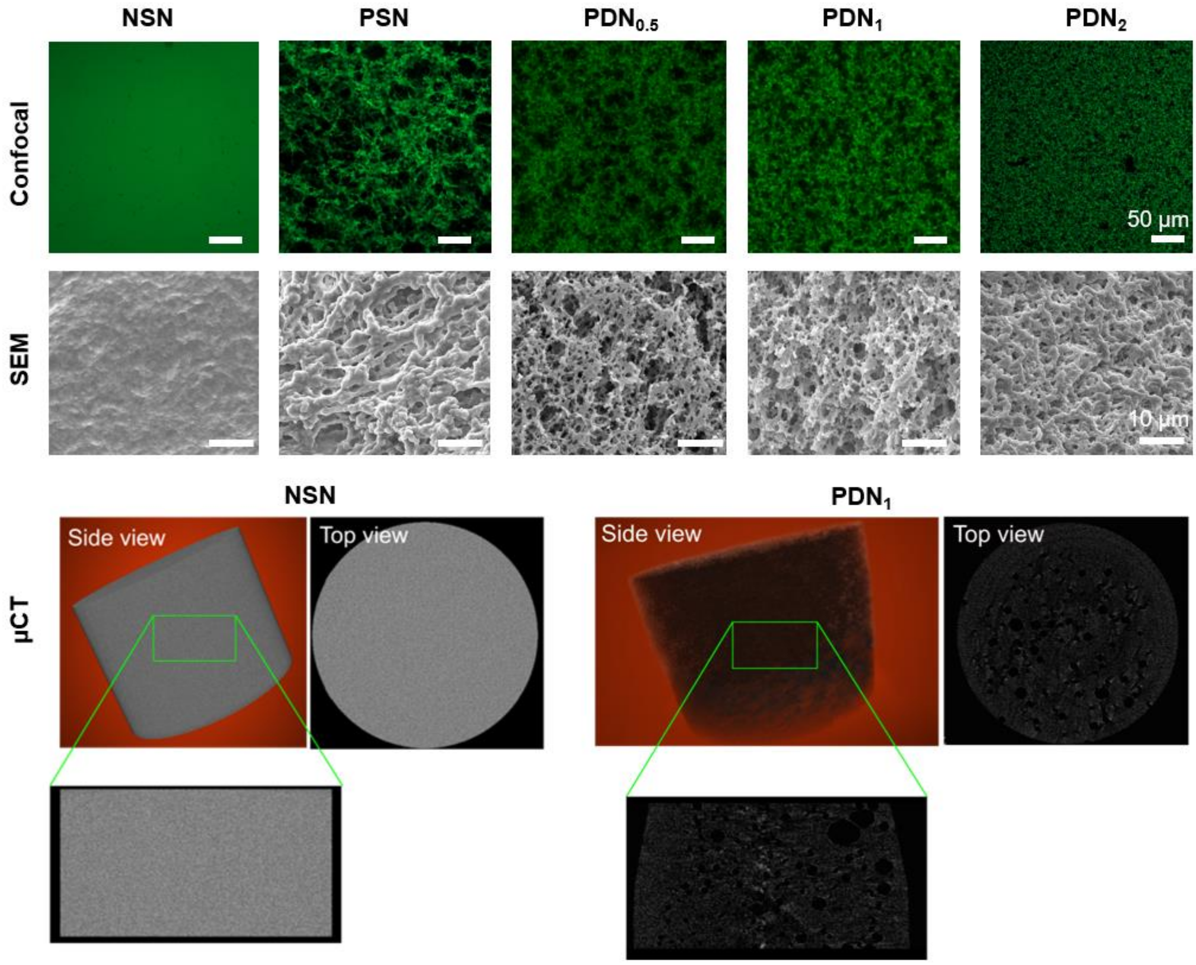

Figure S5. Confocal, SEM, and µCT images showing the porous structures of NSN, PSN, and PDNs. Sample size, $N = 3$.

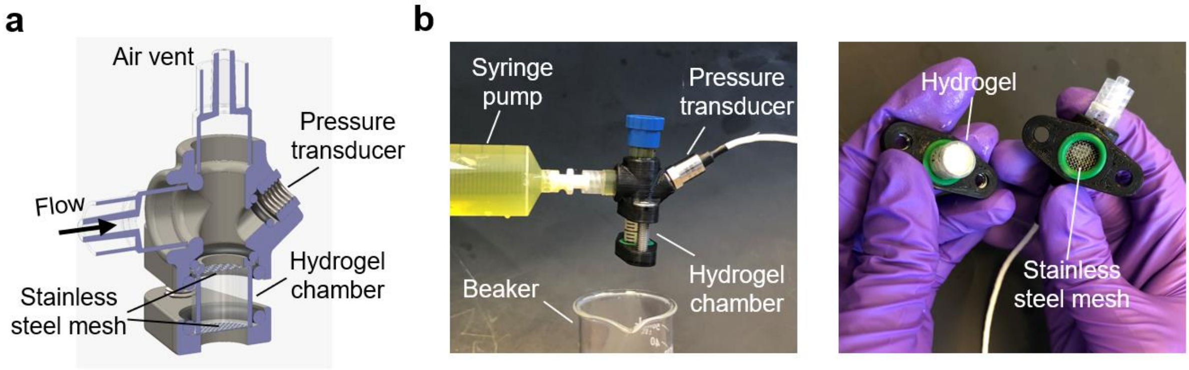

Figure S6. Permeability setup. (a) Schematics showing the internal structure of the perfusion chamber. (b) Digital photos showing the setup of the experiment.

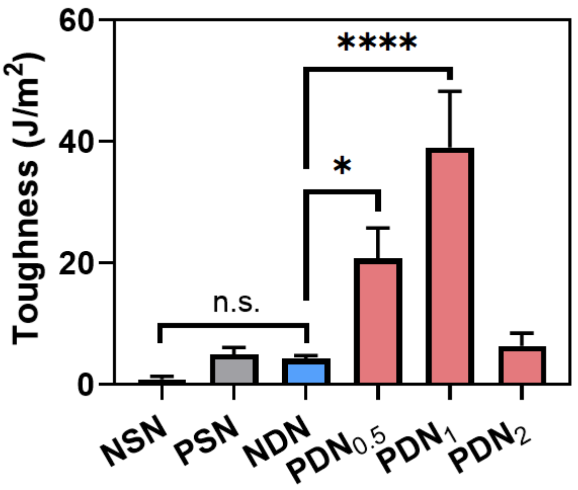

Figure S7. Comparison of toughness among NSN, PSN, NDN, and PDNs.

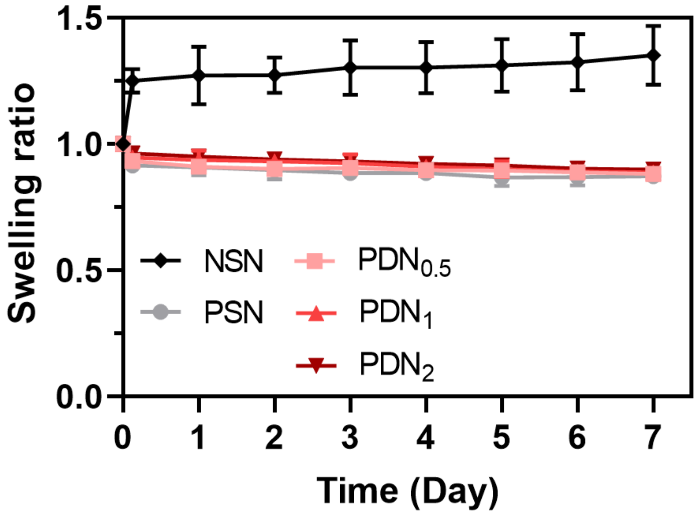

Figure S8. Swelling ratio of different hydrogels immersed in PBS for 7 days. Sample size, *N* = 4.

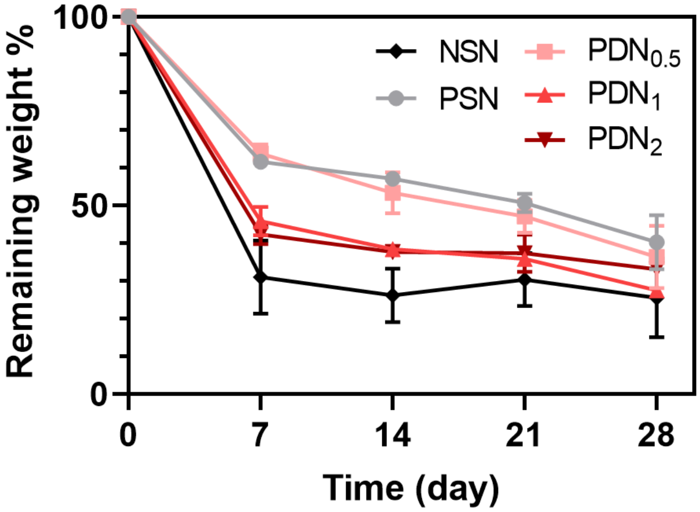

Figure S9. Biodegradation assay showing the remaining weight of different hydrogels when exposed to an enzyme solution over time. Sample size, N = 4.

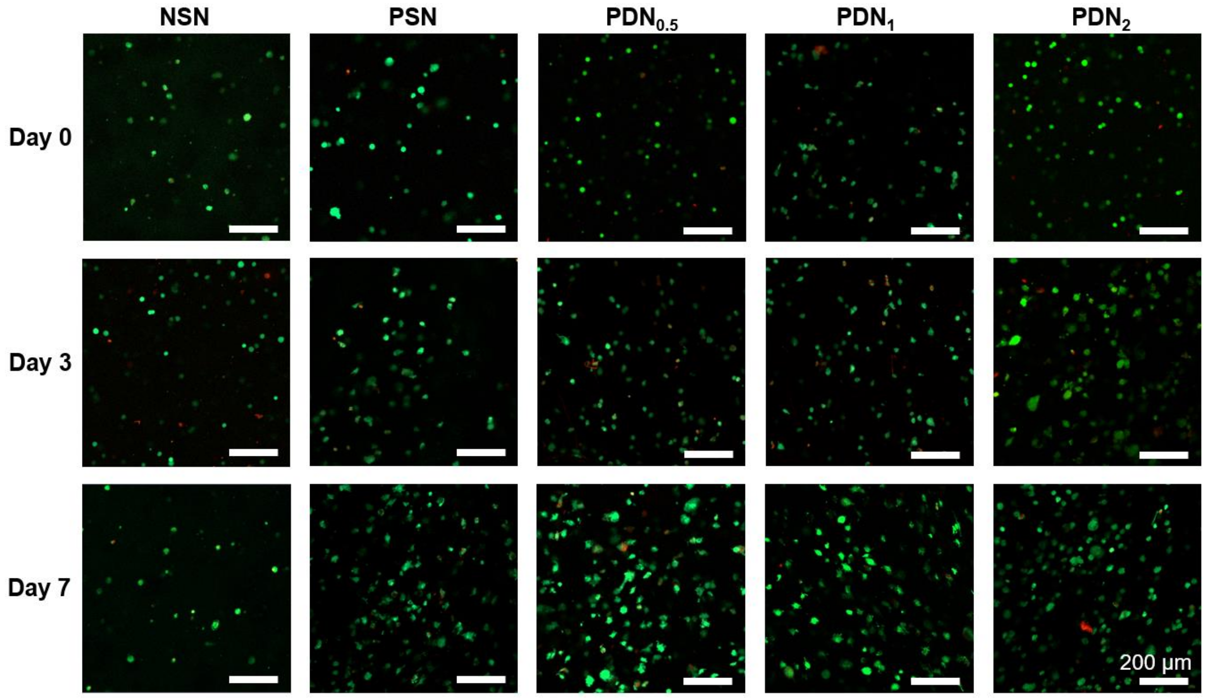

Figure S10. Viability of hVFFs encapsulated inside different hydrogels at Day 0, 3, and 7. Live cells are shown in green and dead cells in red. Sample size, $N = 4$.

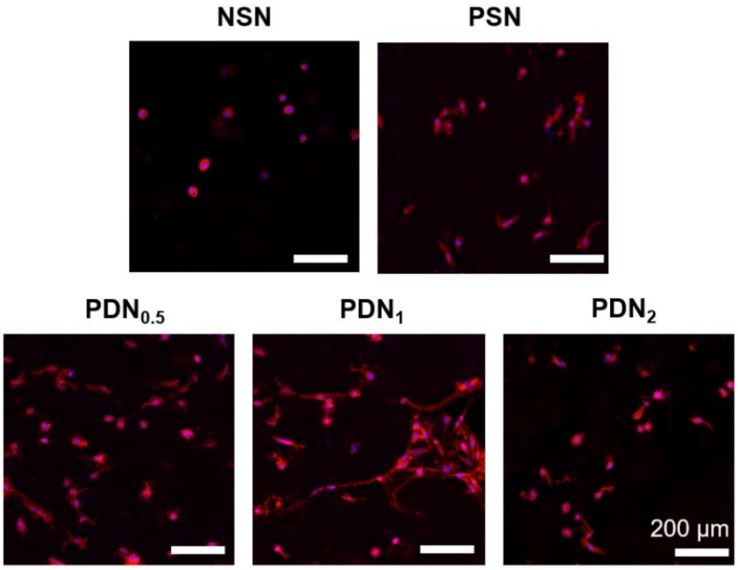

Figure S11. Morphology for hVFFs encapsulated inside different hydrogels at Day 7. F-actin is shown in red and nuclei in blue. Sample size, N = 4.

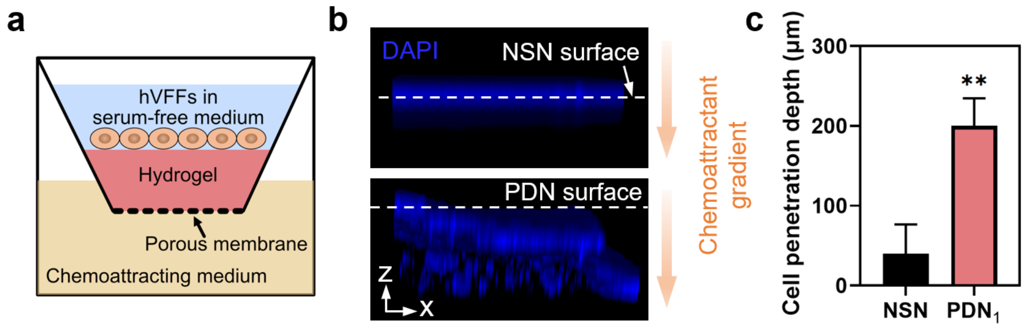

Figure S12. Comparison of cell penetration in NSN and PDN. (a) Experimental setup. (b) Confocal imaging showing hVFFs penetration into different hydrogels. Cells were counterstained with DAPI. (c) Penetration depth of hVFFs into different hydrogels. Sample size, $N = 3$. ** $P < 0.01$.

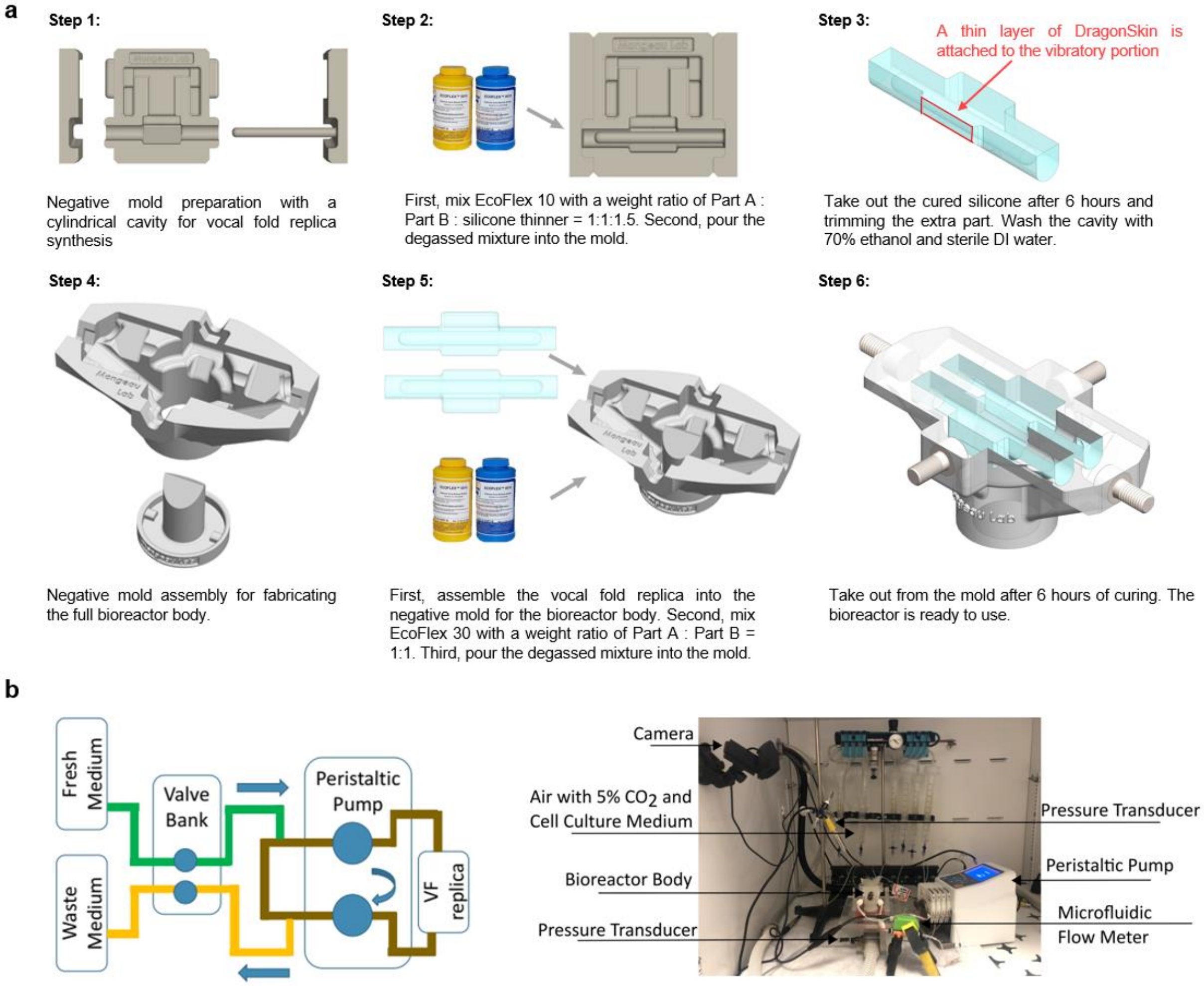

Figure S13. Detailed fabrication process and design for the phonomimetic bioreactor system. (a) Step-by-step instructions on how to fabricate a bioreactor body. (b) Schematic and digital photo showing the configuration of the control loop and arrangement of the complete bioreactor setup.

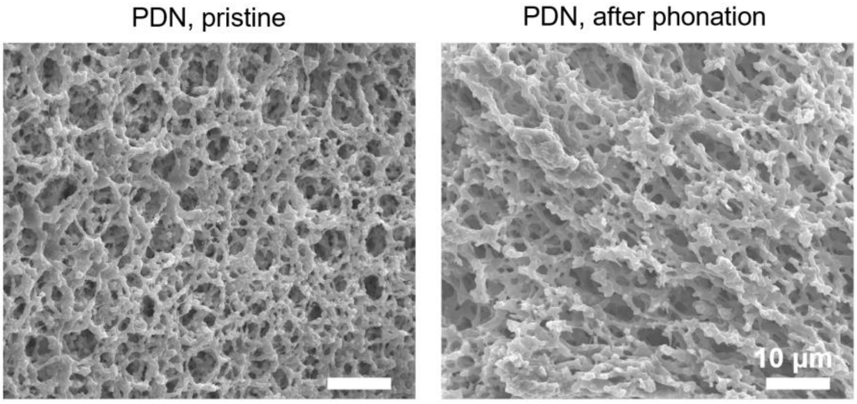

Figure S14. Porous structure of PDN before and after being stimulated inside bioreactor for over 1 million cycles under perfusion.

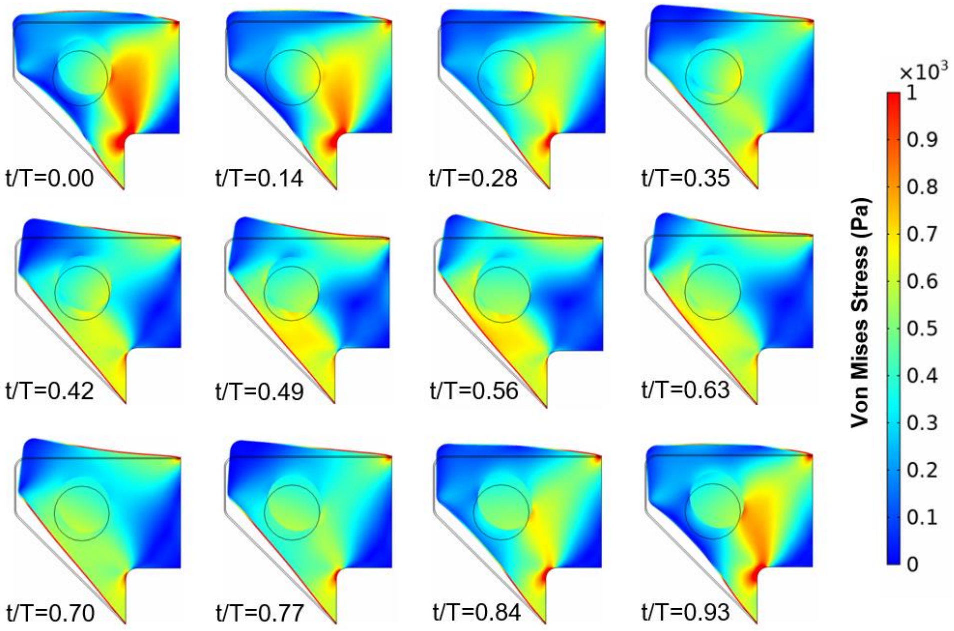

Figure S15. FEA simulation showing the stress distribution of PDN and the elastomeric parts of the bioreactor during one period of oscillation. t/T represents the normalized time during one period. Black contours indicate the undeformed shape and the inner circle refers to the hydrogel.

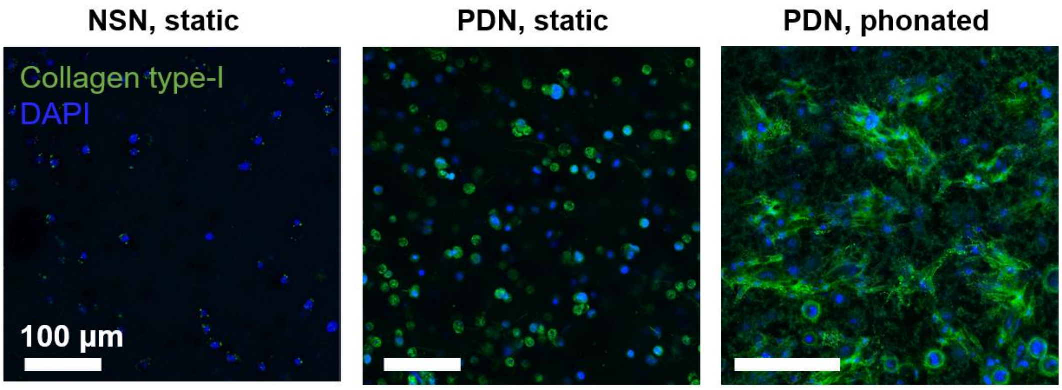

Figure S16. Collagen secretion by hVFFs within different hydrogels with and without mechanical stimulations at Day 7.

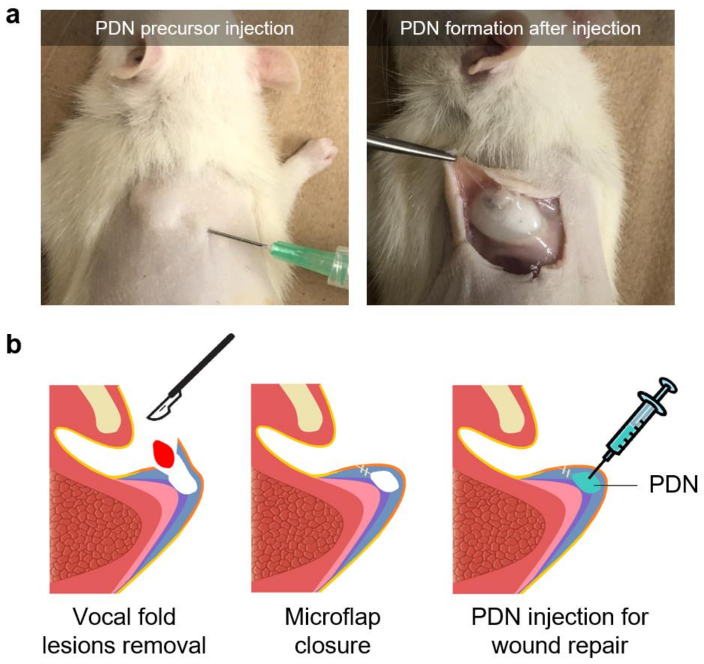

Figure S17. Use of PDN in animals. (a) Injection of PDN (0.85 mL) into a rat cadaver subcutaneously through a fine needle (21G). PDN formed in situ without leakage. (b) Schematic illustration showing the potential use of PDN for vocal fold repair and regeneration.

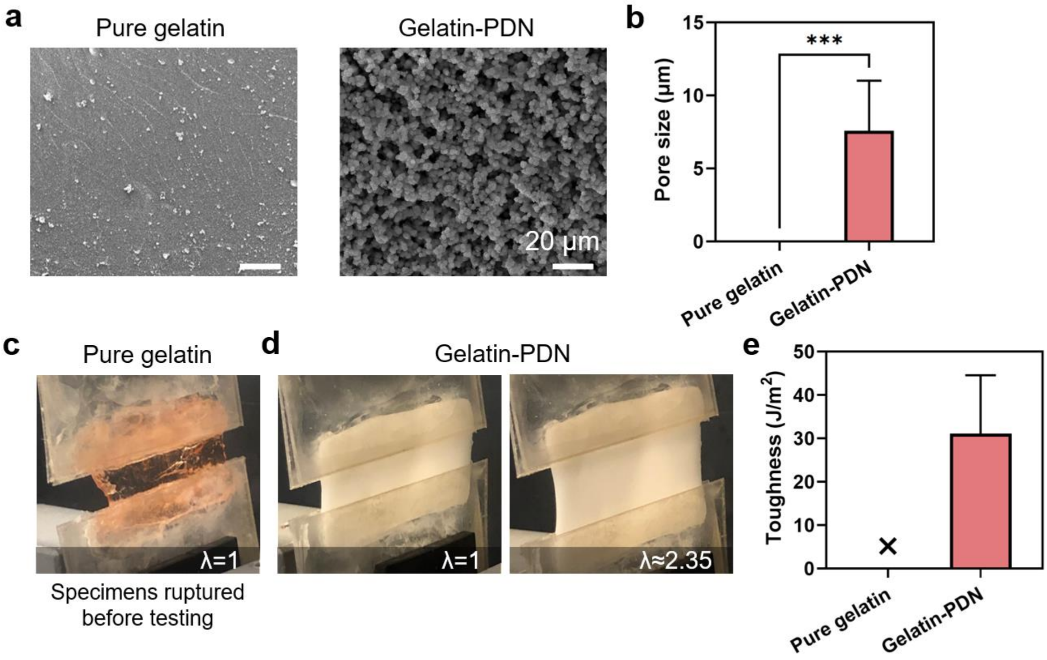

Figure S18. Pore size and toughness of gelatin-based PDN. (a) SEM images showing the structures of pure gelatin and gelatin-PDN. (b) Pore size comparison. (c Photos showing the pure gelatin specimens ruptured during sample preparation steps due to the brittleness of the hydrogel matrix. (d) Stretchability and (e) toughness of gelatin-PDN hydrogels. Toughness of pure gelatin hydrogels was not measured due to specimen rupture before testing and denoted with a cross sign. Sample size, N = 3.

Table S1. Summary of structural and mechanical properties of representative hydrogels and biological tissues.

		Pore size (μm)	Porosity (%)	Toughness Γ (J m^{-2})	Permeability (m^2)	$\tau_{1/2}$ (s)	Cytocompatible synthesis?	Refs.
**Injectable tough hydrogels**	***This work***	6-10	~ 21-54	***Γ***: 5-39<br>***W***[a]: ~ 14<br>***λ***[b]: ~ 3	10^{-14}-10^{-12}	10^{1}-10^{2}	Yes	-
	MethGH-HA hydrogel	N/A[c]	~ 0	***Γ***: *N/A*<br>***W***: 9-14<br>***λ***: ~ 3	N/A	N/A	Yes	[27]
	Dual-click tough hydrogel	N/A	~ 0	N/A	N/A	N/A	Yes	[26]
	PVA-CPBA/Ca	N/A	~ 0	N/A	N/A	N/A	Yes	[29]
	PVA-Bioglass	N/A	N/A	N/A	N/A	N/A	Yes	[55]
	Fibrin-gelatin nanoparticles	~10	~ 35	***Γ***: *N/A*<br>***W***: 9-10<br>***λ***: ~ 1.5	N/A	N/A	Yes	[31]
**Commonly used hydrogels**	Alginate	0.005-0.017	~ 0	1-10	N/A	10^{2}-10^{4}	Yes	[46,56,57]
	Agarose	0.08-0.4	N/A	15	10^{-17}-10^{-16}	10^{2}-10^{3}	Yes	[58–60]
	Chitosan	< 0.1	~ 0	1-10	N/A	10^{4}	Yes	[38]
	Gelatin	0.012-0.03	N/A	0.5-5	10^{-18}-10^{-15}	10^{3}-10^{4}	Yes	[61–64]
	Hyaluronic acid	0.005-0.012	N/A	N/A	N/A	10^{2}-10^{4}	Yes	[65,66]
	PEGDA	0.007-0.025	~ 0	N/A	10^{-17}-10^{-15}	N/A	Yes	[67,68]
	Polyacrylamide	~0.01	~ 0	10-500	10^{-18}-10^{-16}	>10^{4}	No	[46,69]
	Collagen gel	1.1-2.2	N/A	N/A	10^{-16}-10^{-15}	10^{0}-10^{2}	Yes	[70]
**Preformed porous scaffolds**	Bioprinted GelMA	18-53	~ 10-50	N/A	N/A	N/A	Yes	[12]
	Alginate cryogel	~ 30-100	~ 70	N/A	N/A	N/A	No	[11]
	Collagen sponge (freeze-dried)	95-150	> 99	N/A	10^{-13}	N/A	No	[71]
	Bioglass foam	~300	~ 90-95	N/A	10^{-9}	N/A	No	[72]
	Polycaprolactone scaffold	~1000	~ 30-70	N/A	10^{-10}-10^{-8}	N/A	No	[73,74]
**Biological tissues**	Vocal fold	~1-100	~ 90	160-450	10^{-13}-10^{-12}	~60		[75,76]
	Liver	0.1	~ 20	160	10^{-18}-10^{-14}	500		[56,77,78]
	Skin	5-500	~80	1000-20000	10^{-17}-10^{-16}	N/A		[79–81]
	Tendon	4-12	~ 60-70	N/A	10^{-21}-10^{-17}	~1		[81–83]
	Intervertebral disc	0.0015	N/A	N/A	AF: 10^{-17}<br>NP: 10^{-18}	~1 (NP)	Not applicable	[84]
	Bone	6-300	~ 3-80	400-30000	10^{-25}-10^{-10}	N/A		[85,86]
	Small-intestinal submucosa	1-10	~ 87	N/A	10^{-17}	N/A		[86,87]
	Articular cartilage	~0.006	~ 75	690-1300	10^{-17}	1500		[9,88]

[a] ***W***: work of fracture, kJ m^{-3}. [b] ***λ***: Stretchability. [c] N/A: not available.

Table 2. Simulation parameters used in the numerical model

Parameter	Value
Inlet average velocity of air (m s^{-1})	2.66
Outlet pressure	0
Half of the initial glottal gap size (mm)	1
Epithelium thickness (mm)	0.1
Minimum mesh size(mm)	0.0075
Maximum mesh size (mm)	1.33
Dragon Skin density (kg m^{-3})	1 070
Dragon Skin dynamic viscosity (Pa·s)	20
Dragon Skin Young's modulus (Pa)	592 949
Dragon Skin Poisson's ratio	0.49
Dragon Skin isotropic structural loss factor	0.24
Ecoflex 00-10 density (kg m^{-3})	1 040
Ecoflex 00-10 Young's modulus (Pa)	9 693
Ecoflex 00-10 Poisson's ratio	0.49
Ecoflex 00-10 isotropic structural loss factor	0.53

Table 3. Material parameters of the hydrogels used in simulation

Parameter	NSN	PSN	PDN
Density (kg m^{-3})	1 000	1 000	1 000
Poisson's ratio	0.49	0.49	0.49
Ogden parameter α	3.24	1.91	2.79
Ogden parameter μ (Pa)	1 913.57	2 526.60	3 159.89
Mass damping parameter δ (s^{-1})	0.0093	0.027	0.026
Stiffness damping parameter β (s)	0.0029	0.0021	0.0048

Movie S1. Motions of bioreactor during phonomimetic mechanical stimulation.

Movie S2. Finite element simulations showing the stress distributions of injected hydrogels during phonation.

Additional references:

[55] Y. Zhao, Z. Cui, B. Liu, J. Xiang, D. Qiu, Y. Tian, X. Qu, Z. Yang, *Adv. Healthcare Mater.* **2019**, *8*, 1900709.
[56] O. Chaudhuri, L. Gu, D. Klumpers, M. Darnell, S. A. Bencherif, J. C. Weaver, N. Huebsch, H. Lee, E. Lippens, G. N. Duda, D. J. Mooney, *Nature Mater.* **2016**, *15*, 326.
[57] A. D. Augst, H. J. Kong, D. J. Mooney, *Macromol. Biosci.* **2006**, *6*, 623.
[58] H. J. Kwon, A. D. Rogalsky, D.-W. Kim, *Polym. Eng. Sci.* **2011**, *51*, 1078.
[59] E. M. Johnson, W. M. Deen, *AIChE J.* **1996**, *42*, 1220.
[60] P. G. Righetti, B. C. W. Brost, R. S. Snyder, *J. Biochem. Bioph. Meth.* **1981**, *4*, 347.
[61] A. A. Thakre, A. K. Singh, *J. Adhes. Sci. Technol.* **2018**, *32*, 1899.
[62] M. Miller, J. D. Ferry, F. W. Schremp, J. E. Eldridge, *J. Phys. Chem.* **1951**, *55*, 1387.
[63] S. T. Koshy, R. M. Desai, P. Joly, J. Li, R. K. Bagrodia, S. A. Lewin, N. S. Joshi, D. J. Mooney, *Adv. Healthc. Mater.* **2016**, *5*, 541.
[64] A. K. Miri, H. G. Hosseinabadi, B. Cecen, S. Hassan, Y. S. Zhang, *Acta Biomater.* **2018**, *77*, 38.
[65] X. Xu, A. K. Jha, D. A. Harrington, M. C. Farach-Carson, X. Jia, *Soft Matter* **2012**, *8*, 3280.
[66] J. Lou, R. Stowers, S. Nam, Y. Xia, O. Chaudhuri, *Biomaterials* **2018**, *154*, 213.
[67] G. S. Offeddu, E. Axpe, B. A. C. Harley, M. L. Oyen, *AIP Adv.* **2018**, *8*, 105006.
[68] Y.-C. Chiu, M.-H. Cheng, H. Engel, S.-W. Kao, J. C. Larson, S. Gupta, E. M. Brey, *Biomaterials* **2011**, *32*, 6045.
[69] V. Kapur, J. C. Charkoudian, S. B. Kessler, J. L. Anderson, *Ind. Eng. Chem. Res.* **1996**, *35*, 3179.

[70] S. Ramanujan, A. Pluen, T. D. McKee, E. B. Brown, Y. Boucher, R. K. Jain, *Biophys. J.* **2002**, *83*, 1650.
[71] F. J. O'Brien, B. A. Harley, M. A. Waller, I. V. Yannas, L. J. Gibson, P. J. Prendergast, *Technol. Health Care.* **2006**, *15*, 3.
[72] I. Ochoa, J. A. Sanz-Herrera, J. M. García-Aznar, M. Doblaré, D. M. Yunos, A. R. Boccaccini, *J. Biomech.* **2009**, *42*, 257.
[73] A. G. Mitsak, J. M. Kemppainen, M. T. Harris, S. J. Hollister, *Tissue Eng. Part A* **2011**, *17*, 1831.
[74] M. R. Dias, P. R. Fernandes, J. M. Guedes, S. J. Hollister, *J. Biomech.* **2012**, *45*, 938.
[75] C. C. Xu, R. W. Chan, *Tissue Eng. Part A* **2008**, *14*, 1893.
[76] A. K. Miri, L. X. Chen, R. Mongrain, L. Mongeau, *J. Voice* **2016**, *30*, 251.
[77] C. Gokgol, C. Basdogan, D. Canadinc, *Med. Eng. Phys.* **2012**, *34*, 882.
[78] S. Raghunathan, D. Evans, J. L. Sparks, *Ann. Biomed. Eng.* **2010**, *38*, 1789.
[79] Q. Zhang, J. A. Johnson, L. W. Dunne, Y. Chen, T. Iyyanki, Y. Wu, E. I. Chang, C. D. Branch-Brooks, G. L. Robb, C. E. Butler, *Acta Biomater.* **2016**, *35*, 166.
[80] J. B. Holyoke, W. C. Lobitz, *J. Investig. Dermatol.* **1952**, *18*, 147.
[81] R. Oftadeh, B. K. Connizzo, H. T. Nia, C. Ortiz, A. J. Grodzinsky, *Acta Biomater.* **2018**, *70*, 249.
[82] H. Ramakrishna, Biodegradable Biphasic Scaffolds using Braiding Technology and Surface Treatments for Regeneration of Tendon-Bone Junction Tissue. Doctoral thesis, North Carolina State University, Raleigh, North Carolina, **2019**.
[83] S. L. Butler, C. Chen, *Biomed. Eng.* **1997**, 5.
[84] D. H. Cortes, N. T. Jacobs, J. F. DeLucca, D. M. Elliott, *J. Biomech.* **2014**, *47*, 2088.
[85] L. Cardoso, *J. Biomech.* **2013**, 13.
[86] G. A. P. Renders, L. Mulder, L. J. van Ruijven, *J. Anat.* **2007**, *210*, 239.
[87] M. W. Beatty, A. K. Ojha, J. L. Cook, L. R. Alberts, G. K. Mahanna, L. R. Iwasaki, J. C. Nickel, *Tissue Eng.* **2002**, *8*, 955.
[88] C. D. DiDomenico, M. Lintz, L. J. Bonassar, *Nat. Rev. Rheumatol.* **2018**, *14*, 393.
